# Medical and Surgical Cases Occurring in the Sisters of Charity Hospital

**Published:** 1869-11

**Authors:** W. W. Miner

**Affiliations:** Member of the Class


ART. II.—*Medical and Surgical Cases occurring in the Sisters of Charity Hospital.*

Reported by W. W. MINER, A. B , Member of the Class.

PROF. EASTMAN'S SURGICAL CLINIC :

*Case V.*—A child, aged seven months; parents live in Canada ; has doubly cleft upper lip and palate, and is introduced to the hospital by Dr. Storck. The middle portion of lip and bone intervening between the fissures, is triangular in shape, being connected at its apex with the parts above, and measured at its base one-half of an inch in breadth. It projects forward from its normal position, three-fourths of an inch, and is separated latterally from the superior maxillary bones by fissures three-fourths of an inch in breadth, which completely divide the palatal arch. I have never seen sutures connecting an inter-maxillary bone in the human subject, but such a bone exists in some orders of animals. In the present case it is proposed to relieve this deformity, which is seen to be remarkably great, by the approximation of the parts of the upper lips. This involves the removal of the inter-maxillary portion of bone, which, in its forward projection, resists any depression of the parts. The palate is so widely separated that no attempt will be made for obtaining the union of its parts, though the operation called staphyloraphy is more often performed at present than formerly. Prepared specimens of infant and fœtal crania exist, showing an inter-maxillary bone, but they are seldom met with. The age of the child, seven months, is favorable for the operation, which should in all cases be made as soon as the loss of blood incident to its performance can be sustained. The child is enveloped in a sheet, and anæsthesia, more or less perfect, produced. The edges of the parts of the lip involved in the fissures are well trimmed with the scissors, or sharp pointed bistoury, the intervening bone removed with the forceps and the lip separated for some distance from its attachments beneath, so as to distribute the tension over a greater surface when the parts are united by the pin suture. Juxtaposition of the parts of the lip is nicely made, and the patient placed in the hospital ward until the result of the operation is determined.

*Case VI.*—Mrs. H——, aged forty-three, was brought before the class and operated upon by Dr. Miner, who, at Dr. Eastman's request, remarked that the tumor of the breast, which we now show you, has the symptoms, history and appearances of malignant or cancerous disease. It is hard and movable, about two inches in diameter, and has grown within the last five or six months. It is not tender on pressure, but severe lancinating pains pass through it and the breast, so as to disturb, sometimes to prevent, sleep. Respecting the nature of cancerous growths, there is a diversity of opinion among the profession, some maintaining that they are primarily constitutional in character, perhaps hereditary in origin, and that the excision of growths thus caused does not retard their progress or delay the fatal termination. Others believe that the affection is a local one, and that the system is secondarily affected. In the early stages of cancerous growths, it is proper to remove them but after they have made considerable progress, they are apt to reappear after excision. A cancerous cachexia is often recognizable, which forbids surgical interference; and clinical experience shows the folly of late excisions. I am of the opinion that tumors which are cancerous, are so from the beginning, though microscopic examination may not reveal it; and, upon careful weighing of evidence, I believe that benign growths do not degenerate into malignant ones—the matter still, however, remaining an open question among pathologists. In excision, better remove considerable of the surrounding healthy parts than leave any affected tissue to necessitate a repetition of the operation. I have a case where cancer has been removed three times, and though with no more apparent thoroughness in the succeeding operations than at first, still it has not yet returned, a period of four years having elapsed since its last removal. I have also removed growths which had progressed to open cancers; but it is better not to interfere with them. Electricity has been vaunted as able to effect the absorption of tumors, but I have never seen any satisfactory results at all, obtained with this agent; the experiments, however, may not have been sufficiently thorough. The growth having been removed, it presents in appearance an outer fibrous structure enclosing within a colloid substance, mostly clear and of honey yellow color. As to the microscopic appearance of this substance, we are at present unable to speak; but if this is malignant in character, it will probably prove actively so. It is impossible to determine as to the malignancy of these growths in their early stages before operating; and, I may add, that after their removal, it is also often quite difficult to decide as to their true character.

*Case VII.*—Mr. C——, aged fifty-three, has chronic hydrocele, involving both testes, but more particularly developed on the left side, while on the right there is, together with hydrocele, inguinal hernia. The serous fluid of the hydrocele, which is an effusion from, and is enclosed by the tunica vaginalis, increases in amount gradually until surgical treatment is demanded, which effects the withdrawal of the fluid by means of a trocar, generally affording only temporary relief, or establishes adhesion of the walls of the sac, by means of stimulating injections, the effect of which some believe to be rather in reinstating the normal absorption of the secretion of the membrane involved in the sac. In the present case, permanent relief of the hydrocele on the left side will be attempted by the injection into the cavity of the emptied sac of two drachms of tinct. iodine, diluted with water, which, by manipulation, is made to penetrate every part, and will excite adhesive inflammation of the walls of the sac. Care is very necessary, in inserting a trocar, that the canula enters within the cavity of the sac, else serious consequences may result from infiltration into the surrounding tissue; the position, also, of the testis in the sac should be determined, and its puncture avoided. It is best to keep the patient quiet for a day or two after the operation. Since an injection of iodine, on the right side, would cause an inflammation which would very likely involve the hernia, no operation will be made upon that side until the success of the efforts on the other side is established, when inflammation may be induced, and, with care, controlled by means of a seton on the side of the hernial sac.

*Case VII.*—Dr. MINER presented a private patient, a little child, with convergent strabismus. After complete anæsthesia, he divided the internal rectus, the eye immediately assuming position in harmony with the opposite eye. He remarked upon the causes of strabismus—its hereditary origin, and how it was often required. Advised early operation when appearing in young children, urging it upon the ground that vision was much more likely to be restored than after the eye had been long accustomed to disuse. The importance of great care in making the operation was explained—the incision through the conjunctiva to be made transversly rather than horizontally, and of as limited extent as possible, thus preventing the caruncle from falling back and making an unsightly exposure of the sclerotic. The tendon was to be hooked up and divided without any more division of tissue than absolutely necessary to effect the desired object. The operation, if properly made, was capable of obviating a great deformity; but, if carelessly or improperly made, often produced an aggravation of the defect. The results varied greatly in different cases. Perhaps it was impossible to uniformly obtain the best results—some cases, which were operated upon in apparently satisfactory manner, not resulting as favorably as expected. It was, however, generally reasonable to infer, that the unsightly results sometimes witnessed were largely due to defect in the operation. At one time the operation was regarded with great favor, and made everywhere without hesitation—this was upon the first introduction of the operation. It soon, however, fell into disrepute, and for a time was nearly abandoned. It finally has become one of the standard operations in Ophthalmic Surgery, and is to be recommended and practiced, but always with the greatest care. It is made, mainly, to remove deformity, and not with the expectation of restoring vision; if adopted early, it sometimes restores, at least partial, more rarely complete vision.

PROF. ROCHESTER'S MEDICAL CLINIC:

*Case IX.*—Mr. C——, aged twenty-seven, was taken five days since with *mania a potu*, and has also sub-acute gastritis. The fatty alcoholic order of the dram-drinker's breath is recognizable, and his respiration has that character peculiar to persistently intemperate persons. The patient is delirious, talks incoherently, has a flushed face, and does not sleep well. The iris is not contracted, but dilated rather; and the tongue is coated with a light fur, and its tip is dropped, a characteristic sign. Bromide of potassium is given to control the delirium, an ounce of whiskey once in four hours to support the nervous system, and mild aperients to improve the condition of the stomach and alimentary canal. The stimulant is to be withdrawn as soon as the general state of the patient will allow, and tonics substituted for it.

The affections produced by continued excessive use of alcohol, which generally have been included under the one term of delirium tremens, are distinctly of two kinds, viz., *mania a potu* and *delirium ebriosum*. *Delirium ebriosum* occurs as the result of a continued alcoholic debauch, which produces active cerebral congestion. The patient is wild, violent and demonstrative in his actions; and is in a high febrile excitement and delirium. All stimulants should be withheld; and the treatment consists of active cathartics such as calomel and seidlitz powders, nervous sedatives, and general tonics. *Mania a potu* is generally caused by the effort of the drunkard to cease from taking, or by the refusal of his stomach to receive, the accustomed stimulant. The disease is sometimes occasioned by the use of tobacco, by long continued sleeplessness, by business excitement, or by shock of an accident. The nervous system deprived of its usual support suffers severe prostration. The delirium is shown in fears and "horrors" rather than in violent passions; there is also vigilance, sleeplessness, loss of appetite, trembling, furred tongue and an excited pulse. Oftentimes the treatment consists simply in a good nutritious diet. The patient should be kept in a quiet room and have strong, good natured attendants, who should reason with the timid, irrational man, and never alarm and excite him. Nervous and arterial sedatives, stomachics, and mild cathartics may be given. The nervous system, however, requires, at first, its usual stimulus, which should be given in quantities less than is habitual and gradually diminished as the general system recovers from its shock. *Mania a potu* is a condition of exhaustion that will not admit of any depletion. The bromides of ammonium and potassium may be given in doses of twenty grains as a nervous sedative, or valerianate of ammonia used, and, as a rule, opiates in some form. You are liable to get cumulative effects in using opiates, but narcosis is indicated by the contracted pupil before its general effects are obtained. Give sufficient intervals between the doses, and let food be taken in the meantime; always watch the pupil when giving opiates, and also the respiration, and thus avoid cumulative effects. A remedy that has come into vogue within the last five or six years is digitalis. The tincture is given in doses of from one to four drachms once in two or three hours. It acts through the nervous centres to diminish the pulse, and is also a diuretic and diaphoretic eliminative. Dr. Percy, of New York, has received two prizes for essays upon the use of this agent. None more strongly opposed digitalis at first, and the large doses of it that were given in London, than myself, but my subsequent experience in the use of it has been quite happy. A patient who had not been improving was given one drachm of tincture of digitalis, and twenty drops of tinct. capsici, once in two hours; two doses were taken, and, at the time of the third dose, he was sleeping well. In another case in the city, digitalis succeeded where active agents had previously failed. Capsicum is used in large doses in the East Indies in such cases, as a stimulant to the stomach; and in mania, the brain symptoms are thought to be secondary to stomachic ones. In very violent cases, cathartics will soothe the patient; two drops of croton oil will subdue the most burly Irishman; but I must caution you in this treatment not to use it unless the symptoms are violent, and the patient's constitution strong. A few years ago *mania a potu* and delirium were similarly treated, viz., by use of opium, and the patient's usual beverage in doses as large as he would bear, which treatment some survived, while many yielded to fatal narcosis. In London, opium and alcohol are condemned *in toto* in this disease, by most of the recent writers, (see Aitkens'

Pract. Med.,) but without sufficient reason it seems to me. If the stomach is unable to bear opiates, hypodermic injections of one-half a grain of morphine may be given, and in three hours be repeated if necessary. I have treated very many cases of this disease here and in New York, and still hold to the opium and stimulant treatment, when employed judiciously. Codeia has a marked advantage over opium, in that it produces no ill effects upon the brain. I have used doses of one-half to one grain, once in three hours, in a large number of cases, and quite successfully. It can be used where you do not dare to give opium.

Delirium and mania are diseases which affect the spirit drinker, especially those who drink the poorer kinds of spirit, while they very rarely attack the beer drinker, and in France and other vine growing countries they hardly ever occur. Medical men should encourage the culture of the grape, and thus avoid so great prevalence of these diseases. There is no reason why America should not be a vine-growing, wine-producing and consuming country. Legislation seems to be nearly futile in preventing the evils of intemperance. The education of the people to the proper exercise and control of their tastes is the only way to rid the world of these evils.*

*Corrections.—In Prof. Rochester's Clinic, as published in the October number, on the 82d page, 17th line, for weakness read restlessness. In the formula for Sat. Sol Potass. Chlor. ℥ iii., read ℥ viii. There were, likewise, errors in Cases II. and III.

